# Use of Phosphatase and Dehydrogenase Activities in the Assessment of Calcium Peroxide and Citric Acid Effects in Soil Contaminated with Petrol

**DOI:** 10.1515/biol-2020-0002

**Published:** 2020-02-28

**Authors:** Kornel Curyło, Arkadiusz Telesiński

**Affiliations:** 1 Department of Plant Physiology and Biochemistry, West Pomeranian University of Technology in Szczecin, 17 Słowackiego St., 71-434 Szczecin, Poland

**Keywords:** soil enzymatic activity, loamy sand, hydrocarbons, remediation

## Abstract

The objective of the study was to compare the effect of calcium peroxide and citric acid on the activity of acid phosphatase (ACP), alkaline phosphatase (ALP), and dehydrogenases (DHA) in uncontaminated soil and soil contaminated with petrol. The experiment was carried out on samples of loamy sand under laboratory conditions. Petrol was introduced to soil samples at a dose of 0 and 50 g·kg 1 DM, as well as calcium peroxide or citric acid at a dose of 0, 50, 100, or 150 mg·kg 1 DM. The humidity of the samples was brought to 60% maximum water holding capacity, and the samples were incubated at 20°C for 8 weeks. Enzyme activity was determined on days 1, 14, 28, and 56. The obtained results demonstrated that the addition of calcium peroxide and citric acid did not result in significant changes in the activity of the determined enzymes in uncontaminated soil. However, it was observed that the application of calcium peroxide, particularly at the dose of 150 mg·kg 1 DM, largely alleviated the impact of petrol on the enzymatic activity of the soil contaminated with petrol. Moreover, among the determined enzymes, the activity of DHA was found to be the best indicator of the effect of calcium peroxide on the soil ecosystem.

## 1 Introduction

Soil quality is governed mainly by the transformation of organic matter, primarily associated with microorganisms and the enzymes they produce as well as the rate of biogeochemical changes in the circulation of elements [1]. Organic matter is subject to continuous transformations, and its amount and quality depend on the rate of mineralization and humification of organic compounds [2]. Soil enzymes catalyze the transformations associated with matter and the energy transformations in ecosystems. Activity of these enzymes is considered to be one of the more sensitive indicators of their functioning. It reflects both the direction and the nature of the biogeochemical processes, as well as the whole of the basic transformations associated with the biological and physicochemical properties of the soil [3]. Evaluation of the enzymatic activity can provide early evidence of the changes in the soil environment, long before the changes of the chemical composition and physical properties of soils begin [4]. Determination of the quality of soil contaminated with various xenobiotics, as well as forecasting the outcomes of changes occurring in the soil environment, is a difficult task due to the complexity and heterogeneity of this environment [5]. The strong effect of the entire set of parameters determining the soil environment disturbs the individual effects at the level of a single cell, as well as the entire biocenose formed by the populations of various species of soil organisms [6].

One of the most important groups of soil enzymes is dehydrogenases (DHA). They are present in all the live cells of microorganisms [7], and hence are often considered to be the indicators of the general microbial activity of the soil [8]. The importance of DHA in the assessment of soil contamination is furthermore supported by the fact that this group of enzymes lacks the capacity to accumulate in the extracellular environment. The role of DHA involves the biological oxidation of organic matter in soil through the transfer of hydrogen from an organic substrate to inorganic acceptors [9]. Brzezińska et al. [10] determined that these enzymes may use not only oxygen molecules as electron acceptors but also other compounds present in the cells of anaerobic microorganisms. Thus, the activity of DHA reflects the rate of transformations occurring in soil.

Phosphatases form another important group of soil enzymes [5]. They play a significant role in the process of biochemical mineralization of organic phosphorus, and thus may act as a good indicator of the mineralization potential of organic phosphorus and biological activity taking place in soil [11]. Phosphatases form a group of enzymes that hydrolyze esters and the anhydrides of phosphoric acid [12].

Exploitation of crude oil, and in particular failures associated with its extraction, storage, transport, and processing, is the major cause of environmental contamination with oil products. The harmful effect of crude oil primarily stems from its oily consistency because the hydrocarbons present in the oil form a layer on the surface of soil inhibitng the access of oxygen to the lower layers of the soil [13]. Soil aeration is an important factor influencing the survival of a specific population of microorganisms in the given environment, including those participating in the decomposition of petroleum hydrocarbons [9].

One of the promising agents that can be used for the removal of petroleum derivatives from the soil is peroxides [14]. During the decomposition of these compounds, hydrogen peroxide is formed that not only acts as a source of oxygen for the microorganisms capable of biodegrading the petroleum derivatives but also creates hydroxyl radical capable of oxidizing various organic pollutants [15].

Van Hees et al. [16] determined that the fate of hydrophobic organic pollutants may largely depend on the presence of short-chain organic acids. Only in recent years, reports on the use of these substances in remediation processes, in particular phytoremediation of soils contaminated with petroleum derivatives or heavy metals [17, 18], have been published. However, the precise mechanism of action of the short-chain organic acids on the hydrocarbons is yet to be fully explained [19].

Therefore, the objective of the present study was to compare the effect of calcium peroxide and citric acid on the activity of acid phosphatase (ACP), alkaline phosphatase (ALP), and dehydrogenases (DHA) in uncontaminated soil and soil contaminated with petrol.

## 2 Materials and Methods

### 2.1 Experiment design

The soil material was collected from the topsoil of typical brown earths (Brunic Arenosol). According to the graining classification of the US Department of Agriculture, the sample taken was soil with a granulometric composition of loamy sand. The content of particular fractions, expressed in g·kg^1^, was as follows: sand (0.05–2 mm)— 748.6; silt (0.002–0.05 mm)—231.3; and clay (<0.002 mm)— 20.1. The soil contained the following, expressed in g·kg^1^: C_org_—8.71; N_tot_—0.97. The hydrolytic acidity of the soil was 9.9 mmol(+)·kg^1^, and the pH value in 1 M KCl was 6.4. The soil was air-dried and sieved with a mesh of 2 mm.

The experiments were carried out in triplicate under laboratory conditions. Unleaded petrol was introduced to the soil samples weighing 1 kg at doses of 0 and 50 g·kg^1^ DM. The basic properties of petrol are provided in Table 1. Following petrol, calcium peroxide or citric acid was added to the uncontaminated and petrol-contaminated soil at doses of 0, 50, 100, and 150 mg·kg^1^ DM. The humidity of the soil was adjusted to 60% maximum water-holding capacity, and the samples were incubated at 20°C in tightly closed containers for 8 weeks. On days 1, 14, 28, and 56 of the experiment, the activity of ALP [EC 3.1.3.1], ACP [EC 3.1.3.2], and DHA [EC 1.1.1] was determined using spectrophotometry.

**Table 1:** Properties of petrol

Property	Value
Number of carbon atoms	C_6_-C_12_
Density [g·cm^-3^]	0.69
Lower heating value [MJ·kg^-1^]	42.21
Final boiling point [°C]	78
C [%]	84
H [%]	16

### 2.2 Determination of soil enzyme activity

Activity of ALP and ACP was determined according to the method of Tabatabai and Bremener [20]. In this method, a substrate (buffered disodium *p*-nitrophenyl phosphate hexahydrate solution) is added to the soil, and the samples are incubated for 1 h at 37°C. The *p*-nitrophenol (*p*-NP) compound released by the activity of phosphatases is extracted and coloured with sodium hydroxide and determined spectrophotometrically at 400 nm. Activity of phosphatases was calculated based on the calibration curve and was expressed in mg *p*-NP·kg^1^ DM·h^1^.

Activity of DHA was determined according to the method of Casida et al. [21]. This method involves the incubation of soil with a colorless, water-soluble substrate, TTC (2,3,5-triphenyltetrazolium chloride), for 24 h at 25°C. TTC is enzymatically reduced to a colored, water-insoluble product, triphenyl formazan (TPF). By replacing oxygen and other naturally occurring acceptors, TTC overtakes electrons and protons detached by DHA from the oxidized organic compounds. After incubation, TPF is extracted from the soil with ethanol and spectrophotometrically determined at a wavelength of 485 nm. Activity of phosphatases was calculated based on the calibration curve and was expressed in mg *p*-NP·kg^1^ DM·h^1^.

### 2.3 Data analysis

The obtained results were used to calculate the resistance index according to the formula derived by Orwin and Wardle [22]:

$$RS=1-\frac{2\left|D\right|}{C-\left|D\right|}$$

where D is the difference between the activity of enzymes in control soil (*C*) and the activity of enzymes in soil contaminated with petrol or treated with additives. Values of enzyme resistance index remain in the range from −1 to +1. A value of +1 indicates that the contamination has not produced any effect (maximum resistance), whereas the lower values indicate a stronger effect of the contaminant (lower resistance) [22].

*RS* values were statistically determined using the three-way MANOVA with Statistica 13.3 software [23]. This was done by determining the homogeneous groups with a HSD Tukey post-hoc test, at a level of confidence of *p*<0.05. Analyses were performed independently for each day of measurement. Based on the analysis of the effect measure η^2^ by variance analysis, the percentage shares of all the variable factors affecting the activity of enzymes were defined [24]. Changes in the *RS* values caused by petrol and additives were monitored by cluster analysis using the Ward method and squared Euclidean distance [25].

## 3 Results and discussion

Values of resistance indices of ACP, ALP, and DHA in uncontaminated soil after the application of calcium peroxide or citric acid at all doses were in the range of 0.872–0.922, 0.887–0.936, and 0.911–0.974, respectively (Table 2). Such values, close to 1, indicate the minor influence of both calcium peroxide and citric acid on the activity of phosphatases and DHA in soil. As stated by Kumar et al. [26], resistance (capacity of soil to function) may be considered as an indicator of the ecological activity and is associated with soil quality. Moreover, no significant differences in *RS* values were determined between enzymes after the application of calcium peroxide and citric acid at any of the doses.

**Table 2:** Resistance indices (*RS*) of enzymes in uncontaminated soil treated with calcium peroxide or citric acid

Addition	1	14	28	56
Acid phosphatase (ACP)				
50 CaO_2_	0.874 ± 0.035^a^	0.886 ± 0.026^a^	0.934 ± 0.040^a^	0.897 ± 0.032^a^
50 C_6_H_8_O_7_	0.892 ± 0.029^a^	0.908 ± 0.043^a^	0.903 ± 0.017^a^	0.896 ± 0.023^a^
100 CaO_2_	0.894 ± 0.011^a^	0.904 ± 0.028^a^	0.906 ± 0.034^a^	0.922 ± 0.025^a^
100 C_6_H_8_O_7_	0.907 ± 0.013^a^	0.883 ± 0.044^a^	0.901 ± 0.021^a^	0.898 ± 0.020^a^
150 CaO_2_	0.909 ± 0.027^a^	0.916 ± 0.027^a^	0.917 ± 0.027^a^	0.916 ± 0.021^a^
150 C_6_H_8_O_7_	0.915 ± 0.026^a^	0.904 ± 0.040^a^	0.896 ± 0.022^a^	0.902 ± 0.020^a^
Alkaline phosphatase (ALP)				
50 CaO_2_	0.896 ± 0.011^a^	0.903 ± 0.024^a^	0.887 ± 0.034^a^	0.907 ± 0.018^a^
50 C_6_H_8_O_7_	0.887 ± 0.041^a^	0.894 ± 0.022^a^	0.883 ± 0.018^a^	0.904 ± 0.047^a^
100 CaO_2_	0.911 ± 0.032^a^	0.914 ± 0.043^a^	0.921 ± 0.040^a^	0.899 ± 0.025^a^
100 C_6_H_8_O_7_	0.897 ± 0.043^a^	0.906 ± 0.030^a^	0.904 ± 0.028^a^	0.894 ± 0.034^a^
150 CaO_2_	0.914 ± 0.024^a^	0.920 ± 0.022^a^	0.936 ± 0.019^a^	0.914 ± 0.024^a^
150 C_6_H_8_O_7_	0.903 ± 0.033^a^	0.908 ± 0.018^a^	0.914 ± 0.033^a^	0.907 ± 0.042^a^
Dehydrogenases (DHA)				
50 CaO_2_	0.935 ± 0.054^a^	0.928 ± 0.028^a^	0.964 ± 0.047^a^	0.945 ± 0.018^a^
50 C_6_H_8_O_7_	0.925 ± 0.042^a^	0.911 ± 0.019^a^	0.936 ± 0.051^a^	0.947 ± 0.050^a^
100 CaO_2_	0.942 ± 0.022^a^	0.938 ± 0.049^a^	0.948 ± 0.074^a^	0.941 ± 0.038^a^
100 C_6_H_8_O_7_	0.931 ± 0.028^a^	0.919 ± 0.033^a^	0.966 ± 0.049^a^	0.952 ± 0.041^a^
150 CaO_2_	0.948 ± 0.028^a^	0.941 ± 0.029^a^	0.946 ± 0.017^a^	0.943 ± 0.053^a^
150 C_6_H_8_O_7_	0.927 ± 0.041^a^	0.920 ± 0.051^a^	0.974 ± 0.077^a^	0.944 ± 0.029^a^

The same letters for an enzyme in columns are assigned to the same homogeneous groups (HSD Tukey test); the significance of the letters is *p*<0.05; data are presented as mean values ± SD; 50, 100, and 150 determine the doses of additives expressed in mg·kg^-1^ DM; CaO_2_ calcium peroxide, C_6_H_8_O_7_ citric acid

After the application of petrol on day 1 of the experiment, the *RS* values of ACP, ALP, and DHA were calculated to be 0.772, 0.599, and 0.543, respectively. On the subsequent days of measurement, the *RS* values of all the determined enzymes decreased. The *RS* values of ALP and DHA remained constant until the end of the experiment, whereas the values of ACP on the last day of the experiment were similar to those on day (Table 3). The lowest *RS* value was noted on day 14 for phosphatases (ACP 0.179; ALP 0.209), and on day 28 for DHA (0.283). Such low *RS* values indicate the disturbance of biological balance under the impact of petrol [22]. This confirms the results reported by numerous authors, who have indicated the negative impact of petrol on the activity of various enzymes, for example, dehydrogenases [8, 9, 27, 28], phosphatases [27, 28, 29, 30], catalase [9], β-glucosidase [28], peroxidases [9], urease [27, 31], and nitrate reductase [32]. Oversaturation of soil with petrol considerably restricts the development or disturbs the survival of numerous soil microorganisms [33]. Borowik at al. [34] showed that soil contamination with petroleum increased of operational taxonomic unit numbers were demonstrated for *Actinobacteria* and *Acidobacteria*. According to Gałązka et al. [35], soil samples contaminated with hydrocarbons were colonized mainly by *Alphaproteobacteria*, *Betaproteobacteria*, and *Gammaproteobacteria*, which were strongly correlated with the biological activity of these soils. The prevailing classes included also *Actinobacteria* and *Acidobacteria*. The high counts of bacteria classified to *Proteobacteria*, *Bacteroidetes*, and *Actinobacteria* in the soils polluted with petroleum products were also confirmed by results of investigations conducted by Yan et al. [36], Hou et al. [37], and Jung et al. [38]. These taxa, potentially capable of degrading alkanes diminished the diversity of microorganisms [34]. However, Afzal et al. [39] reported that the soils contaminated with hydrocarbons were colonized mainly by *Pseudomonaceae*, *Burkholderiaceae*, *Bacillaceae*, and *Enterobacteriaceae*. Changes in microbiological activity of a soil heavily contaminated with hydrocarbons stems primarily from the disturbance of trophic conditions and oxygen availability. Excess of active forms of organic carbon results in the deficiency of nitrogen, phosphorus, and oxygen [40].

**Table 3:** Resistance indices (*RS*) of enzymes in petrol-contaminated soil treated with calcium peroxide or citric acid

Addition	1	14	28	56
Acid phosphatase (ACP)				
Petrol	0.772 ± 0.069^a^	0.179 ± 0.014^d^	0.306 ±0.027^c^	0.791 ± 0.040^c^
50 CaO_2_	0.789 ± 0.026^a^	0.186 ± 0.015^d^	0.308 ± 0.023^c^	0.814 ± 0.026^bc^
50 C_6_H_8_O_7_	0.792 ± 0.017^a^	0.198 ± 0.016^d^	0.315 ± 0.012^c^	0.794 ± 0.024^c^
100 CaO_2_	0.790 ± 0.038^a^	0.276 ± 0.009^bc^	0.330 ± 0.013^c^	0.868 ± 0.014^ab^
100 C_6_H_8_O_7_	0.793 ± 0.038^a^	0.255 ± 0.010^c^	0.328 ± 0.024^c^	0.817 ± 0.022^bc^
150 CaO_2_	0.806 ± 0.012^a^	0.417 ± 0.021^a^	0.439 ± 0.009^a^	0.895 ± 0.020^a^
150 C_6_H_8_O_7_	0.801 ± 0.014^a^	0.315 ± 0.015^b^	0.378 ± 0.024^b^	0.824 ± 0.007^bc^
Alkaline phosphatase (ALP)				
Petrol	0.599 ± 0.040^a^	0.209 ± 0.016^b^	0.373 ± 0.025^a^	0.400 ± 0.038^a^
50 CaO_2_	0.601 ± 0.010^a^	0.220 ± 0.019^ab^	0.387 ± 0.033^a^	0.403 ± 0.004^a^
50 C_6_H_8_O_7_	0.601 ± 0.009^a^	0.212 ± 0.013^ab^	0.380 ± 0.011^a^	0.408 ± 0.010^a^
100 CaO_2_	0.621 ± 0.012^a^	0.225 ± 0.013^ab^	0.406 ± 0.016^a^	0.417 ± 0.015^a^
100 C_6_H_8_O_7_	0.604 ± 0.013^a^	0.216 ± 0.009^ab^	0.398 ± 0.011^a^	0.409 ± 0.017^a^
150 CaO_2_	0.624 ± 0.013^a^	0.262 ± 0.010^a^	0.421 ± 0.016^a^	0.419 ± 0.016^a^
150 C_6_H_8_O_7_	0.618 ± 0.027^a^	0.229 ± 0.022^ab^	0.406 ± 0.013^a^	0.416 ± 0.021^a^
Dehydrogenases (DHA)				
Petrol	0.643 ± 0.035^a^	0.376 ± 0.026^c^	0.283 ± 0.034^c^	0.395 ± 0.037^b^
50 CaO_2_	0.643 ± 0.019^a^	0.377 ± 0.030^c^	0.306 ± 0.009^c^	0.399 ± 0.018^b^
50 C_6_H_8_O_7_	0.658 ± 0.014^a^	0.379 ± 0.019^c^	0.291 ± 0.011^c^	0.397 ± 0.020^b^
100 CaO_2_	0.662 ± 0.017^a^	0.442 ± 0.026^b^	0.419 ± 0.022^b^	0.451 ± 0.019^b^
100 C_6_H_8_O_7_	0.670 ± 0.030^a^	0.390 ± 0.011^c^	0.300 ± 0.011^c^	0.411 ± 0.028^b^
150 CaO_2_	0.665 ± 0.017^a^	0.486 ± 0.022^a^	0.520 ± 0.042^a^	0.638 ± 0.038^a^
150 C_6_H_8_O_7_	0.667 ± 0.011^a^	0.405 ± 0.026^c^	0.315 ± 0.017^c^	0.413 ± 0.018^b^

The same letters for an enzyme in columns are assigned to the same homogeneous groups (HSD Tukey test); the significance of the letters is *p*<0.05; data are presented as mean values ± SD; 50, 100, and 150 determine the doses of additives expressed in mg·kg^-1^ DM; CaO_2_ calcium peroxide, C_6_H_8_O_7_ citric acid

The process of spontaneous purification of a soil contaminated with petroleum derivatives is often prolonged and is influenced by both the course of spontaneous physicochemical reactions and the occurrence of indigenous living organisms exhibiting specific enzymatic activities [41]. It is often necessary to introduce modifying additives to the soil environment, which will influence the process of degradation of the petroleum-derived hydrocarbons [42].

After the application of calcium peroxide and citric acid at the dose of 50 mg·kg^1^ DM to the soil contaminated with petrol, no significant changes in *RS* values were observed throughout the experiment. Application of citric acid at higher doses resulted in a significant increase of *RS* values on certain days as compared with soil contaminated with petrol without any additives added, but this was observed only for ACP. After the application of citric acid at a dose of 100 mg·kg^1^ DM, an increase in *RS* value to 0.076 was observed on day 14 and at the dose of 150 mg·kg^1^ DM on days 14 and 28 (by 0.136 and 0.072, respectively). Our previous study on the influence of low-molecular-weight organic acids (oxalic acid, tartaric acid, and citric acid) on the phosphatase activity in soil contaminated with creosote showed the highest efficiency of citric acid [19]. In addition, Huang et al. [43] demonstrated that the presence of low-molecular-weight organic acids in soil influences its sorption of ACP. Furthermore, An et al. [44] stated the efficiency of distinct organic acids in the reduction of polycyclic aromatic hydrocarbons (PAHs) sorption in the soil as follows: citric acid > oxalic acid > tartaric acid > lactic acid > acetic acid. It should be emphasized at this point that citric acid is one of the intermediates of the Krebs cycle. Biodegradation of *n*-alkanes, which are the major components of petrol, primarily involves their oxidation to suitable fatty acid, which is then subject to β-oxidation, resulting in the generation of acetyl-CoA. Subsequently, acetyl-CoA participates in the cycle of tricarboxylic acids through the process of oxygen respiration [45].

However, compared to the soil contaminated with petrol, the application of calcium peroxide at the dose of 100 mg·kg^1^ DM increased the *RS* values of ACP on the day 14 (0.097) and that of DHA on the days 14 and 28 (0.066 and 0.136, respectively). The dose of 150 mg·kg^1^ DM of calcium peroxide added to soil was found to be the most efficient in alleviating the effect of petrol on the enzymatic activity of the soil. Increased *RS* values were found for ACP on days 14, 28, and 56 (0.238, 0.133, and 104, respectively), for ALP on day 14 (0.053), and for DHA on days 14, 28, and 56 (0.110, 0.237, and 0.243, respectively). A stimulating effect of calcium peroxide on the activity of various enzymes was determined in the soil contaminated with diesel oil [14], coal tar creosote [46], phenanthrene [15], and anthracene [47]. As stated by Małachowska-Jutsz and Niesler [15], chemical oxidation with the use of calcium peroxide is a promising degradation method for a wide range of organic compounds. The latest research has further demonstrated that chemical oxidation may co-occur with oxygen metabolism in soil, and such combination is a more efficient solution in comparison with the natural bioremediation processes [48, 49, 50]. Calcium peroxide is identified to be one of the most efficient agents for the degradation of petroleum-derived hydrocarbons [51], polycyclic aromatic hydrocarbons [52], tetrachloroethylene [53], and polychlorinated biphenyls (PCBs) [54].

The effect of both citric acid and calcium peroxide varied on different days of measurement. This was demonstrated by the analysis of η^2^. The percentage contribution of this factor in the formation of activity of dehydrogenases and phosphatases in soil contaminated with petrol was definitely the highest (Table 4). Previous research also demonstrated the time-variable effect of various additives modifying the enzymatic activity of soil contaminated with petroleum derivatives [8, 14, 15, 19, 46, 55].

**Table 4:** Percentage share of observed variability factors η^2^

Variable factor	ACP	ALP	DHA
Kind of additive (K)	0.24	0.10	5.07
Dose od additive (D)	1.80	0.46	6.51
Day of experiment (T)	95.87	97.47	75.11
K × D	0.25	0.03	3.92
K × T	0.13	0.03	3.08
D × T	0.96	0.08	2.21
K × D × T	0.10	0.04	2.06
Error	0.64	1.78	2.04

ACP acid phosphatase, ALP alkaline phosphatase, DHA dehydrogenases

**Figure 1:**
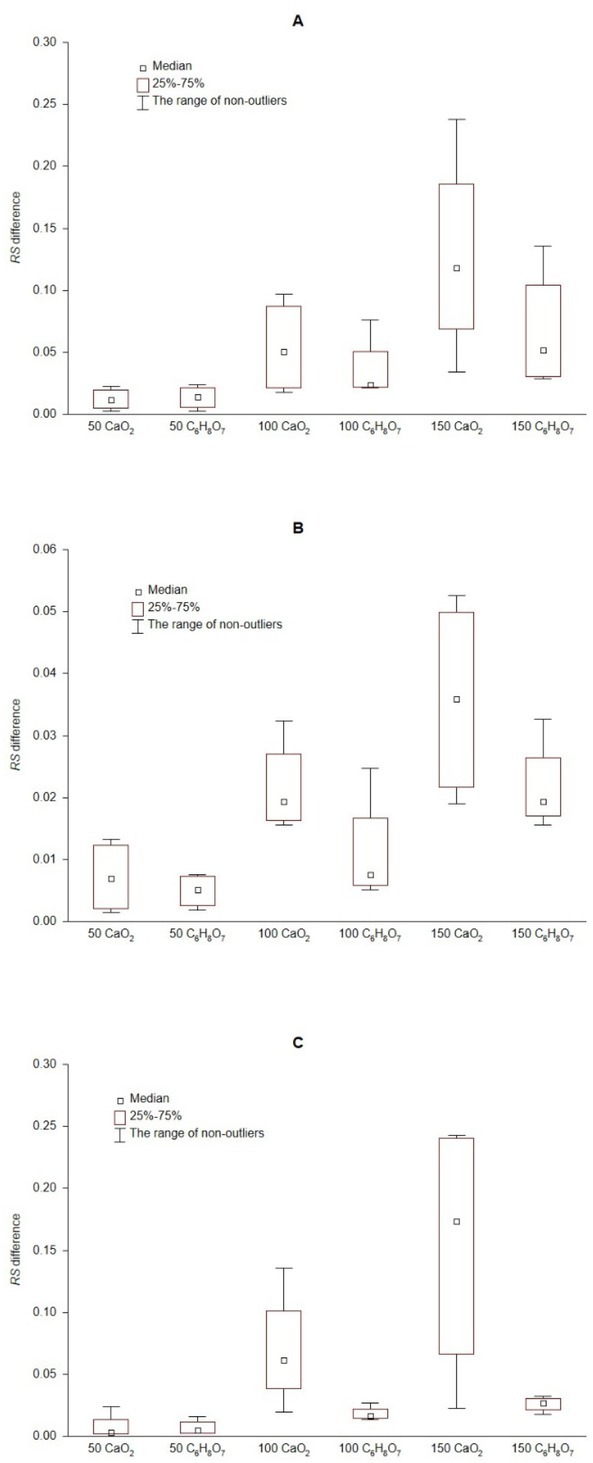
Median of *RS* differences for activity of acid phosphatase (A), alkaline phosphatase (B), and dehydrogenases (C) in petrol-contaminated soil treated with calcium peroxide or citric acid and petrol-contaminated soil; 50, 100, and 150 determine the doses of additives expressed in mg·kg^-1^ DM; CaO_2_ calcium peroxide, C_6_H_8_O_7_ citric acid

The higher efficiency of calcium peroxide in alleviating the influence of petrol on the activity of soil dehydrogenases and phosphatases has been confirmed by the analysis of the median of differences between the *RS* values in soil contaminated with petrol and modifying agents added and the *RS* values in soil contaminated only with petrol. For all the determined enzymes, the median of *RS* values increased with the increase of the dose of modifying additives. In addition, for all the enzymes, the calculated differences were considerably greater after the introduction of calcium peroxide than those found with citric acid (Figure 1). The comparison of differences in *RS* values further allowed determining that the efficiency of the enzymes in the course of remediation under the influence of calcium peroxide, as well as citric acid, was in the following order: DHA > ACP > ALP. Kaczyńska et al. [8] demonstrated that DHA are a very good indicator of soil contamination with petroleum derivatives, as well as the efficiency of the conducted remediation. This is due to the fact that this group of enzymes constitutes an integral part of the undisturbed microorganism cells, thus indicating the biological activity of the soil [9]. Moreover it is well known that some microbes in soil have the potential to greatly enhance the rate of organic P (P_o_) or inorganic (P_i_) cycling (i.e. by solubilizing insoluble organic- and mineral-bound P) [56]. In soils, concentrations of available P in soil solution are typically low due to the comparatively low content of P in the parent material, but also due to the high reactivity of P_i_ that results in strong retention by the soil’s mineral matrix. This has led to microorganisms developing a wide range of strategies to enhance P availability in soil. Although plants can only take up P_i_, fungi and bacteria can also potentially take up low molecular weight (LMW) P_o_ [57].

**Figure 2:**
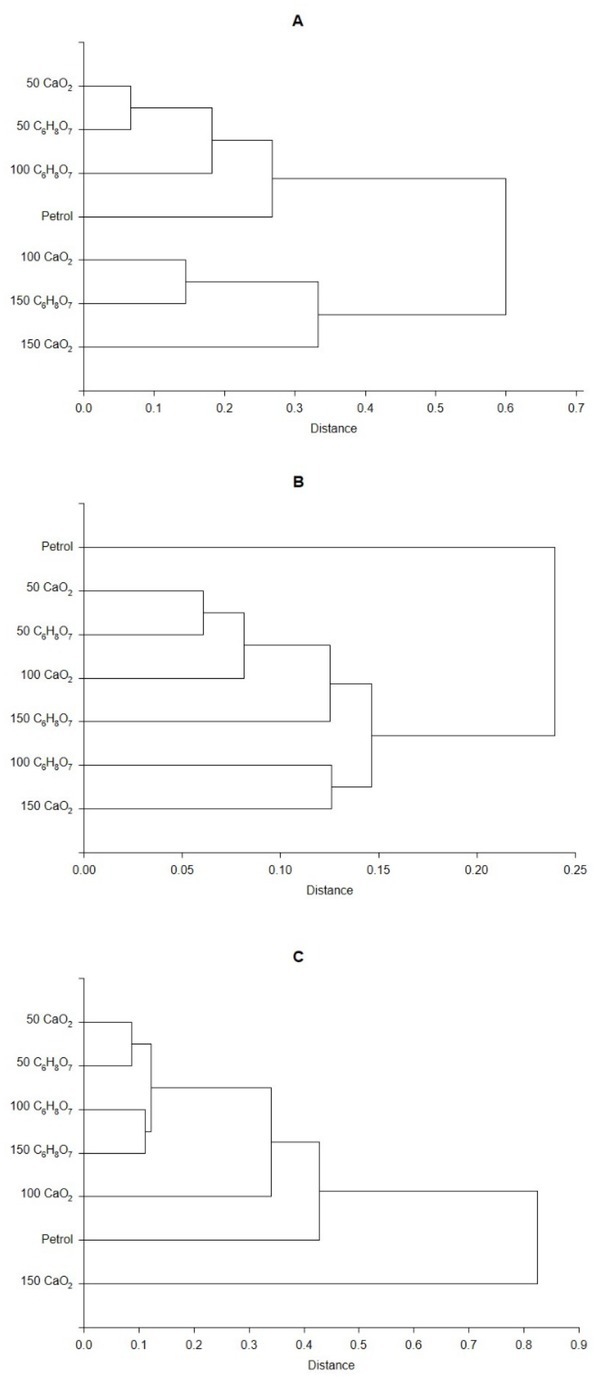
Cluster analysis dendrograms of *RS* values in petrol-contaminated soil treated with calcium peroxide or citric acid and petrol-contaminated soil; 50, 100, and 150 determine the doses of additives expressed in mg·kg^-1^ DM; CaO_2_ calcium peroxide, C_6_H_8_O_7_ citric acid

However, interpretation of the dendrograms obtained from cluster analysis demonstrated that it is difficult to unambiguously indicate the type and dose of an additive having the greatest impact on the activity of ALP. However, for ACP the greatest impact of citric acid was found at the dose of 150 mg·kg^1^ DM and calcium peroxide at the dose of 100 and 150 mg·kg^1^ DM, whereas for DHA the greatest impact of calcium peroxide was found at the dose of 150 mg·kg^1^ DM (Figure 2).

## 4 Conclusions

Currently, the search for efficient methods of remediation of soils contaminated with petroleum derivatives constitutes one of the most important ecotoxicological issues. We found that addition of citric acid did not demonstrate any considerable impact on the alleviation of the toxic effect of petrol on the activity of phosphatases and dehydrogenases in contaminated soil. However, chemical oxidation after the introduction of calcium peroxide, primarily at a dose of 150 mg·kg^1^ DM, leveled the toxic influence of petrol on the activity of ACP and DHA. Moreover, it can be stated that DHA are the best indicator of the effect of hydrogen peroxide on the biochemical activity of a soil contaminated with petrol. The results obtained are currently of great importance because of the ongoing research concerning encapsulation technologies of calcium peroxide with other materials and the preparation of calcium peroxide nanoparticles. Synthesis of calcium peroxide in nano size by increased ratio of surface to volume can increase the speed of Fenton reaction during bioremediation of soil contaminated with hydrocarbons.
